# Educational Interventions for Labor Epidural Awareness in the United States: Protocol for a Scoping Review

**DOI:** 10.2196/85380

**Published:** 2026-07-08

**Authors:** David Basulto, Jacqueline Gamboa, Guadalupe Vazquez-Perez, Vidhani Goel, Roberto Sagaribay, Jyoti Desai, Kavita Batra

**Affiliations:** 1 Department of Medical Education, Kirk Kerkorian School of Medicine at the University of Nevada, Las Vegas, 1701 W. Charleston Blvd., Suite 200-05, Las Vegas, NV, 89102, United States, 1 702 823 3751; 2 Office of Research, Kirk Kerkorian School of Medicine at the University of Nevada, Las Vegas, Las Vegas, NV, United States; 3 School of Public Health, Kirk Kerkorian School of Medicine at the University of Nevada, Las Vegas, Las Vegas, NV, United States; 4 Department of Gynecologic Surgery and Obstetrics, Kirk Kerkorian School of Medicine at the University of Nevada, Las Vegas, Las Vegas, NV, United States

**Keywords:** labor epidural, neuraxial anesthesia, educational interventions, labor pain management, awareness, patient education, prenatal care, perinatal care, health disparities

## Abstract

**Background:**

Labor pain is among the most intense forms of pain, and neuraxial analgesia, including epidural, spinal, and combined spinal-epidural techniques, is considered the gold standard for its management. Despite its effectiveness, persistent misconceptions, cultural barriers, and disparities in awareness contribute to underuse among certain populations. Educational interventions have been developed to address these gaps, yet a comprehensive synthesis of such efforts in the United States is lacking.

**Objective:**

This scoping review aims to map the extent, range, and nature of current literature describing educational interventions designed to improve knowledge, awareness, and acceptance of neuraxial analgesia during labor among pregnant women.

**Methods:**

Following the PRISMA-ScR (Preferred Reporting Items for Systematic Reviews and Meta-Analyses extension for Scoping Reviews) framework, peer-reviewed studies published in English were identified through PubMed, Embase, and Scopus from inception to August 2025. Eligible studies include those focusing on pregnant individuals eligible for neuraxial analgesia who received educational or counseling interventions during prenatal or perinatal care. Extracted data include study design, intervention type, delivery method, timing, and outcomes of interest, including patient understanding of risks and benefits, awareness of neuraxial analgesia options, acceptance of or preference for neuraxial analgesia, satisfaction with education, and uptake during labor. Data will be synthesized descriptively using summary tables and figures, with a narrative synthesis to categorize interventions by type, timing, and delivery method and identify patterns and gaps across studies.

**Results:**

This project was conceived in August 2024, and the protocol was registered in the Open Science Framework in January 2025. The database search was conducted in August 2025. As of protocol submission in October 2025, full-text screening and data extraction are pending and are scheduled for May 2026 to June 2026. Data synthesis and drafting of the results will be completed in September 2026. The manuscript will be submitted in October 2026.

**Conclusions:**

This scoping review will map existing educational interventions related to neuraxial labor analgesia and identify gaps in the current literature. The findings may help inform future research and the development of more consistent and accessible patient education approaches.

## Introduction

Labor pain is widely considered one of the most intense forms of pain, and effective pain management is a central component of obstetric care [1]. Neuraxial analgesia, including epidural, spinal, and combined spinal-epidural techniques, is the most effective and widely used method for managing labor pain and is considered the gold standard [2,3]. Professional guidelines from the American College of Obstetricians and Gynecologists recommend offering neuraxial analgesia at any stage of labor, and a patient’s request alone is sufficient indication in the absence of contraindications [1,4]. Despite its effectiveness, use remains variable across populations in the United States, with disparities in access and uptake reported [5,6].

Persistent misconceptions surrounding neuraxial analgesia contribute to variability in use. These include beliefs that it increases cesarean delivery rates, prolongs labor, or leads to long-term complications [7]. Additional barriers such as cultural differences, mistrust, limited health literacy, and language discordance further influence decision-making and access to care [6,8,9].

Importantly, disparities in labor analgesia use should be considered within the broader context of inequities in maternal health outcomes in the United States. Black women in particular experience disproportionately higher rates of maternal morbidity and mortality and may face systemic barriers to equitable care, including differences in access, communication, and trust in the health care system [6,8,9]. These structural and social factors may contribute to differences in awareness, counseling, and uptake of neuraxial analgesia. For example, Hispanic women have been reported to be less likely to receive regional analgesia compared to women of other racial and ethnic backgrounds [8], potentially reflecting gaps in access to education and communication [9].

The decision to focus on the United States was based on the unique structural, cultural, and health care system factors that influence obstetric care delivery and maternal health disparities in this country, including documented inequities in access to labor analgesia among racial and ethnic minority populations. Additionally, educational practices, patient counseling approaches, and labor analgesia use patterns may differ internationally because of variations in health care systems, cultural norms, insurance structures, and obstetric care models. Limiting this review to the United States allows for greater contextual consistency and a more focused evaluation of educational interventions within a shared health care environment.

Ensuring that patients are adequately informed about neuraxial analgesia options is therefore essential to support informed decision-making and patient autonomy. Patient education has been associated with improved understanding, reduced anxiety, and greater satisfaction with the childbirth experience [2]. There is emerging evidence suggesting that structured educational interventions may improve knowledge and acceptance of neuraxial analgesia. For instance, a randomized controlled trial demonstrated that a culturally sensitive intervention combining video, written materials, and in-person counseling increased epidural uptake among Hispanic women and improved understanding across groups [9].

The timing and delivery of education may also influence its effectiveness. Prenatal education has been associated with improved knowledge retention and decision-making compared to intrapartum counseling, when pain and stress may limit comprehension [10]. Additionally, multimodal approaches incorporating visual, written, and verbal formats have been shown to better address diverse learning needs [11].

Despite these findings, there remains a lack of comprehensive synthesis of educational interventions specifically aimed at improving understanding and uptake of neuraxial labor analgesia. Prior reviews have focused on clinical techniques or broader analgesia strategies without addressing patient education or decision-making processes [12,13]. A clearer understanding of how educational interventions are designed, delivered, and evaluated is needed.

Therefore, we propose a scoping review to map the current literature on educational interventions aimed at improving patient understanding and uptake of neuraxial analgesia during labor in the United States. This review will characterize intervention types, delivery methods, and reported outcomes and identify gaps in the literature to inform future research.

## Methods

### Ethical Considerations

Because this study does not involve human participants or patient-level data, institutional ethics approval is not required. This protocol was developed in accordance with the PRISMA-P (Preferred Reporting Items for Systematic Reviews and Meta-Analyses Protocols) reporting guidelines, and the completed scoping review will be conducted and reported following the PRISMA-ScR (Preferred Reporting Items for Systematic Reviews and Meta-Analyses extension for Scoping Reviews) framework to ensure methodological transparency and rigor [14,15]. The protocol has been registered with the Open Science Framework. Any important protocol amendments will be documented with the date of modification and description of the change in the final review manuscript.

### Review Questions

Our review questions are as follows:

1. What types of educational interventions delivered by health care professionals during prenatal care have been implemented to increase knowledge, awareness, or acceptance of neuraxial analgesia (epidural, spinal, or combined spinal-epidural) among pregnant women?
2. In what prenatal or perinatal care settings in the United States have these educational interventions been delivered, and how have their effectiveness and outcomes been evaluated?

### Eligibility Criteria

**Figure 1.**
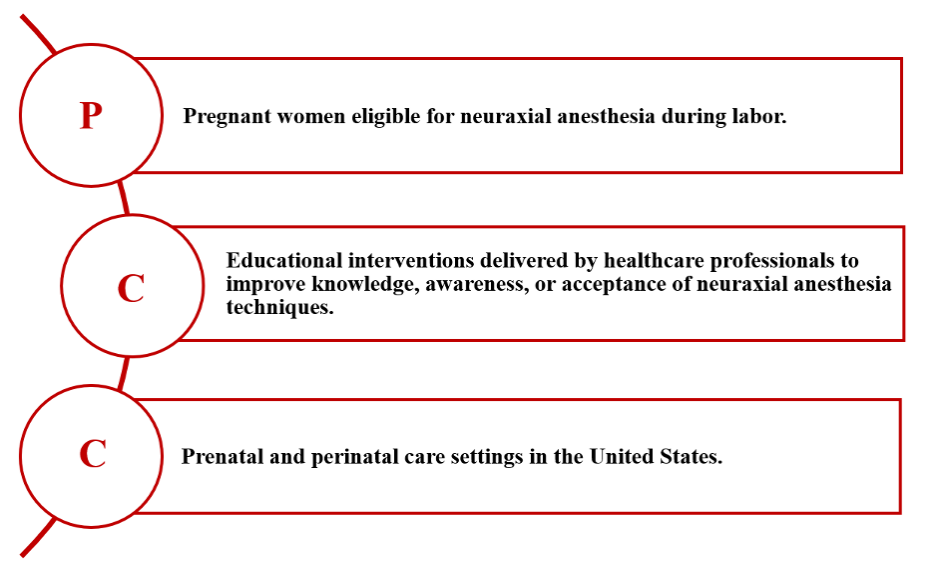
Population, concept, and context framework.

The population, concept, and context (PCC) framework (Figure 1) was used to establish eligibility criteria for this scoping review. The population of interest is pregnant women eligible for neuraxial analgesia during labor. The concept focuses on educational interventions delivered by health care professionals during prenatal care to improve knowledge, awareness, or acceptance of neuraxial analgesic techniques, including epidural, spinal, and combined spinal-epidural analgesia. The context is perinatal care settings in the United States. Eligible studies include peer-reviewed original research published in English using observational or experimental designs and reporting outcomes related to knowledge, awareness, or acceptance of neuraxial analgesia. Excluded studies include reviews, editorials, commentaries, letters, conference abstracts without full manuscripts, non–peer-reviewed publications, articles without available full text, studies unrelated to obstetric populations, and animal studies.

### Informational Sources, Search Strategy, and Screening

The review will use PubMed, Scopus, and Embase as the primary data sources to provide broad coverage of biomedical, interdisciplinary, and public health literature relevant to obstetric analgesia and patient education. The search strategy was developed using the PCC framework to align database searches with the review objectives. Both controlled vocabulary terms (eg, MeSH [Medical Subject Headings] and Emtree terms) and free-text keywords were used to maximize search sensitivity. Search terms included concepts related to pregnant women, neuraxial analgesia, epidural analgesia, spinal analgesia, combined spinal-epidural analgesia, educational interventions, prenatal education, counseling, and labor analgesia. Boolean operators and subject headings were adapted for each database. Elements of the PRISMA-S (Preferred Reporting Items for Systematic Reviews and Meta-Analyses literature search extension) guidance were considered to improve the transparency and reproducibility of the literature search methodology [16]. Table 1 summarizes the PCC-guided search concepts, representative search terms, and database-specific search strategies used in this review. All retrieved records will be imported into Rayyan (Rayyan Systems, Inc) for deduplication and screening. After deduplication, titles, abstracts, and full texts will be independently reviewed by 2 researchers according to the inclusion and exclusion criteria. Disagreements will be resolved through discussion or consultation with a third reviewer. The reference lists of included studies may also be reviewed to identify additional relevant articles.

**Table 1.** Population, concept, and context (PCC)–guided search concepts and database-specific search strategies used for identification of studies related to educational interventions for neuraxial labor analgesia^a^.

PCC component and objective, database, and search strategy	Records retrieved, n
Population: pregnant women eligible for neuraxial labor analgesia^b^	
PubMed 2: “Pregnant Women”[MeSH^c^ terms]; Filters: “English,” “Humans,” and “Female”	7804
Embase 2: (“pregnant women”/exp OR “pregnant women”) AND “human”/de AND [female]/lim AND [english]/lim AND “article”/it	16,995
Scopus 1: TITLE-ABS-KEY (“pregnant women” OR “pregnant people”) AND LIMIT-TO (LANGUAGE, “English”) AND LIMIT-TO (DOCTYPE, “ar”)	4929
Concept: neuraxial labor analgesia^d^	
PubMed 4: (“Anesthesia, Epidural”[MeSH Terms] OR “Analgesia, Epidural”[MeSH Terms]); Filters: “English,” “Humans,” and “Female”	3148
Embase 4: (“epidural analgesia”/exp OR “neuraxial analgesia”/exp OR “labor analgesia”/exp) AND “human”/de AND [female]/lim AND [english]/lim AND “article”/it	219,221
Scopus 3: TITLE-ABS-KEY (“epidural analgesia” OR “neuraxial analgesia” OR “labor analgesia”) AND LIMIT-TO (LANGUAGE, “English”) AND LIMIT-TO (DOCTYPE, “ar”)	5809
Educational intervention: prenatal education and counseling interventions^e^	
PubMed 3: (“Health Education”[MeSH Terms] OR “Prenatal Education”[MeSH Terms] OR “Counseling”[MeSH Terms]); Filters: “English,” “Humans,” and “Female”	45,196
Embase 3: (“health education”/exp OR “prenatal education”/exp OR “counseling”/exp OR “antenatal education”/exp) AND “human”/de AND [female]/lim AND [english]/lim AND “article”/it	232,687
Scopus 2: TITLE-ABS-KEY (“prenatal education” OR “prenatal counseling” OR “health education”) AND LIMIT-TO (LANGUAGE, “English”) AND LIMIT-TO (DOCTYPE, “ar”)	6266
Context: labor and perinatal care settings^f^	
Embase 8: (“childbirth”/exp OR “labor”/exp) AND “human”/de AND [female]/lim AND [english]/lim AND “article”/it	106,892
Scopus 5: TITLE-ABS-KEY (“labor” OR “childbirth”) AND LIMIT-TO (LANGUAGE, “English”) AND LIMIT-TO (DOCTYPE, “ar”)	391,748
Combined search strategy: educational interventions for neuraxial labor analgesia among pregnant women^g^	
PubMed 7: 2 AND 3 AND 4	1
Embase 9: 2 AND 3 AND 4 AND 8	53
Scopus 7: 3 AND 6	8

a The search strategy was developed using the PCC framework and incorporated both controlled vocabulary terms (eg, MeSH [Medical Subject Headings] and Emtree terms) and free-text keywords. Database-specific adaptations and Boolean operators were used as appropriate. Elements of the PRISMA-S (Preferred Reporting Items for Systematic Reviews and Meta-Analyses literature search extension) guidance were considered to improve the transparency and reproducibility of the literature search methodology.

b Representative search terms: “Pregnant Women”[MeSH], “pregnant women,” “pregnant people,” and “obstetric patients.”

c MeSH: Medical Subject Headings.

d Representative search terms: “epidural analgesia,” “neuraxial analgesia,” “spinal analgesia,” “combined spinal-epidural analgesia,” and “labor analgesia.”

e Representative search terms: “prenatal education,” “antenatal education,” “counseling,” “patient education,” and “educational intervention.”

f Representative search terms: “labor,” “childbirth,” “obstetric care,” “perinatal care,” and “maternity care.”

g Representative search terms: combined PCC concepts using Boolean operators.

### Data Sources, Variables, and Data Extraction

Information extracted from each study will include the author or authors, year of publication, study location and setting, study design, participant characteristics such as age and parity, eligibility for neuraxial analgesia, type and method of education delivery, timing of the intervention, educational content, and materials used. Outcomes such as knowledge, awareness, acceptance, patient satisfaction, and uptake of neuraxial analgesia will also be recorded, along with the key findings and reported limitations.

### Quality or Risk-of-Bias Assessment

Because this is a scoping review, a formal risk-of-bias assessment will not be conducted.

### Data Analysis and Presentation

The data extracted from the selected studies will be summarized in tables and charts that reflect the review objectives and eligibility criteria. Expected findings will include details such as study design; year of publication; geographic setting; study population characteristics; type of educational intervention; delivery method; and outcomes related to knowledge, awareness, or acceptance of neuraxial analgesia. Educational interventions will be categorized according to intervention modality (eg, written, verbal, digital, or multimedia approaches), timing of delivery (prenatal vs intrapartum), delivery setting, and personnel involved in delivering the intervention. Outcomes will be mapped descriptively across domains, including knowledge, awareness, acceptance, satisfaction, and uptake of neuraxial analgesia. In addition to descriptive tabulation, a narrative synthesis approach will be used to identify recurring themes, implementation patterns, variations in intervention design, and gaps in the literature across studies.

### Dissemination

Findings will be disseminated through submission to a peer-reviewed journal and presentation at relevant academic and professional conferences.

## Results

**Figure 2.**
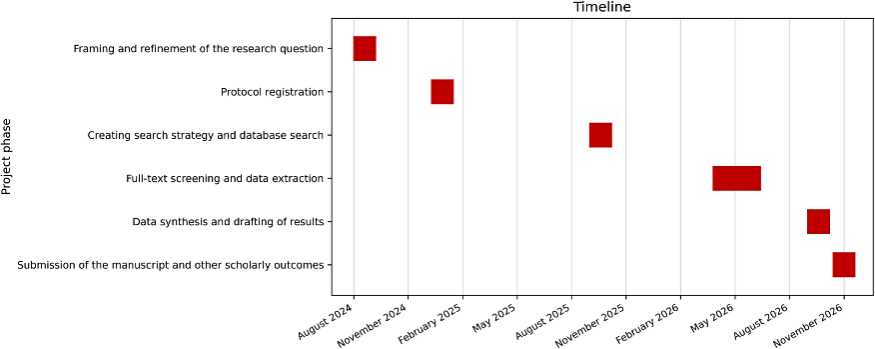
Gantt chart.

This project was conceived in August 2024, and the protocol was registered in the Open Science Framework in January 2025. The database search was conducted in August 2025. As of protocol submission in October 2025, full-text screening and data extraction are pending and are scheduled for May 2026 to June 2026. Data synthesis and drafting of the results will be completed in September 2026. The manuscript will be submitted in October 2026 (Figure 2).

The review will map the extent, range, and nature of research on educational interventions for pregnant women who are eligible to undergo neuraxial analgesia during labor. It will summarize the types of educational interventions that have been implemented and the delivery methods used. It will describe the outcomes and evaluation measures that have been used to assess these interventions and identify the settings in which they have been delivered. The review will also highlight gaps in the current educational interventions and propose areas for future research.

## Discussion

### Expected Findings

This scoping review will map the existing literature and characterize the range, design, and delivery of educational interventions and how outcomes such as knowledge, awareness, and uptake are reported across studies. Existing literature suggests that multimodal, culturally sensitive, and linguistically appropriate approaches may play a role in supporting patient understanding and informed decision-making [1,2,4,9,11].

In relation to intervention format, this review will examine how different educational modalities, including visual, written, and verbal components, are used and described in the literature. Prior research in maternal health literacy suggests that layered, culturally relevant formats may improve comprehension and decision-making confidence, particularly for patients with limited English-language proficiency or lower baseline health literacy [9,11,17]. The role of trusted messengers such as bilingual nurses or community health workers in health literacy will be explored in relation to addressing persistent misconceptions about neuraxial analgesia [7,9].

With respect to timing, this review will assess how educational interventions are delivered across the prenatal and intrapartum periods. Existing studies suggest that prenatal education may be associated with improved knowledge retention and reduced anxiety compared to intrapartum counseling alone [10]. Additionally, prior findings indicate that patients who receive limited or rushed counseling during labor may report lower satisfaction and less clarity in decision-making [18]. These observations highlight areas that this review will further contextualize.

This review will also examine how structural and systemic factors intersect with educational interventions. Existing literature highlights persistent barriers, including language discordance, provider bias, and limited access to interpreter services, that may influence use patterns regardless of educational exposure [6,8,9,19]. This review will explore how studies address these factors and whether interventions are implemented alongside broader institutional support.

In addition to traditional approaches, this review will map the use of digital technologies as emerging modalities for patient education. Mobile apps, SMS text messaging–based platforms, and video modules have been described as potential tools to expand access, particularly in rural or resource-limited settings [20]. The literature also suggests that their effectiveness may depend on accessibility considerations, including digital literacy, language options, and integration with in-person care [9,11].

Finally, this review will identify gaps in the current literature. Prior studies have frequently focused on short-term outcomes such as immediate knowledge or uptake, with limited evaluation of longer-term outcomes, including patient satisfaction, perceived autonomy, or postpartum well-being [2]. Additionally, certain populations—including Black, Indigenous, Asian American, and rural communities—remain underrepresented in many studies despite ongoing disparities in maternal health outcomes [6,8,9,19]. By mapping these gaps, this review may help inform future research and support the development of more consistent and contextually appropriate educational approaches.

### Limitations

This scoping review will have several limitations. First, heterogeneity among the included studies is anticipated due to differences in research design, intervention type, participant populations, and outcome measures. Such variability may limit the ability to make direct comparisons across interventions and prevent generalizable conclusions. Second, although steps will be taken to ensure rigor, there is an inherent risk of reviewer bias. To mitigate this, we will maintain transparency in our methodology, engage subject matter experts throughout the review process, apply standardized quality assessment tools, and perform sensitivity analyses where feasible to account for the influence of lower-quality studies. Third, despite implementing a comprehensive search strategy across multiple databases, it remains possible that some relevant studies may be missed, particularly unpublished or nonindexed literature. This could result in publication bias favoring studies with positive findings. Finally, many outcomes of interest such as trust, perceived respect, satisfaction, and feelings of autonomy are subjective in nature and may not be consistently measured across studies. As a result, our synthesis will rely on both quantitative and qualitative evidence to present the most complete picture possible while recognizing these interpretive constraints.

An additional limitation is that some educational practices may not be captured in the published literature. In many clinical settings, patient education occurs through routine clinical interactions such as anesthesia consultations in the labor and delivery unit, which may be effective but are not consistently evaluated using standardized or measurable tools. Because this review relies on published studies, it may underrepresent these informal or institution-specific educational approaches, which could contribute to gaps between reported interventions and real-world practice.

### Conclusions

This scoping review will provide a structured overview of existing educational interventions designed to improve understanding and uptake of neuraxial labor analgesia in the United States. By systematically mapping key characteristics of these interventions, including modality, timing, delivery setting, and target populations, this review will describe the range and nature of approaches currently represented in the literature. It will also identify patterns in how outcomes such as knowledge, awareness, acceptance, satisfaction, and uptake are defined and measured across studies.

The findings may help inform future research aimed at optimizing patient education strategies and improving accessibility of information for diverse populations. In addition, this review will highlight gaps in the existing literature to identify where further investigation is needed, including the representation of underserved populations, variability in educational approaches, and inconsistencies in outcome reporting.

Overall, this work aims to support the development of more consistent and contextually appropriate educational interventions while providing a foundation for future research and evidence synthesis in this area.

## Supplementary material

10.2196/85380 Checklist 1 PRISMA-P Checklist.

## Abbreviations

MeSH: Medical Subject Headings
PCC: population, concept, and context
PRISMA-P: Preferred Reporting Items for Systematic Reviews and Meta-Analyses Protocols
PRISMA-S: Preferred Reporting Items for Systematic Reviews and Meta-Analyses literature search extension
PRISMA-ScR: Preferred Reporting Items for Systematic Reviews and Meta-Analyses extension for Scoping Reviews
