# Supplementary material for: Educational Interventions for Labor Epidural Awareness in the United States: Protocol for a Scoping Review
Source: JMIR Res Protoc. 2026 Jul 8;15:e85380. doi: 10.2196/85380 (PMC13344085; doi:10.2196/85380)
Supplement: Checklist 1 [file resprot-v15-e85380-s001.docx]

**PRISMA-P 2015 Checklist for Review Protocol**

Educational Interventions for Labor Epidural Awareness: A Scoping Review Protocol

| Section/topic | Item | Checklist item | Reported | Location in manuscript |
| --- | --- | --- | --- | --- |
| Title | 1a | Identify the report as a protocol | Yes | Title page |
| Title | 1b | If update of previous review, identify as such | N/A | Not an update |
| Registration | 2 | Provide registry name and registration number | Yes | Abstract; Methods – Protocol Registration |
| Authors | 3a | Provide author names, affiliations, and emails | Yes | Title page |
| Authors | 3b | Describe author contributions | Yes | Author Contributions section |
| Amendments | 4 | State plan for documenting protocol amendments | Partial | Add brief statement in Methods |
| Support | 5a | Indicate sources of financial or other support | Yes | Funding section |
| Support | 5b | Provide sponsor/funder name | Yes | Funding section |
| Support | 5c | Describe role of sponsor/funder | Yes | Funding section |
| Introduction | 6 | Describe rationale for the review | Yes | Introduction |
| Introduction | 7 | State review objectives/questions | Yes | Review Question section |
| Methods | 8 | Specify eligibility criteria | Yes | Eligibility Criteria |
| Methods | 9 | Describe information sources | Yes | Information Sources/Search Strategy |
| Methods | 10 | Present draft search strategy | Yes | Table 1 |
| Study records | 11a | Describe data management | Yes | Search Strategy and Screening |
| Study records | 11b | Describe selection process | Yes | Search Strategy and Screening |
| Study records | 11c | Describe data collection process | Yes | Data Extraction section |
| Data items | 12 | List and define variables for extraction | Yes | Data Sources, Variables and Data Extraction |
| Outcomes | 13 | Define outcomes and prioritization | Yes | Abstract and Data Extraction |
| Risk of bias | 14 | Describe risk of bias assessment | Yes | Quality or Risk of Bias Assessment |
| Data synthesis | 15a | Describe criteria for quantitative synthesis | N/A | Scoping review; no meta-analysis planned |
| Data synthesis | 15b | Planned summary measures and methods | N/A | Not applicable |
| Data synthesis | 15c | Additional analyses | N/A | Not applicable |
| Data synthesis | 15d | Describe planned synthesis approach | Yes | Data Analysis and Presentation |
| Meta-bias | 16 | Specify assessment of meta-bias(es) | N/A | Not applicable for scoping review |
| Confidence in cumulative evidence | 17 | Describe assessment of strength of evidence | N/A | Not applicable for scoping review |

Reference: Moher D, Shamseer L, Clarke M, Ghersi D, Liberati A, Petticrew M, Shekelle P, Stewart LA. Preferred Reporting Items for Systematic Review and Meta-Analysis Protocols (PRISMA-P) 2015 statement. Syst Rev. 2015;4(1):1.
